# Starving out brain tumors: a reprogrammed lysine catabolism serves as a novel target for glioblastoma treatment

**DOI:** 10.1038/s41392-023-01616-z

**Published:** 2023-10-09

**Authors:** Désirée Gül, Oliver H. Krämer, Christoph Reinhardt

**Affiliations:** 1 Molecular and Cellular Oncology, Department of Otorhinolaryngology Head and Neck Surgery, University Medical Center, Johannes Gutenberg-University Mainz, 55131 Mainz, Germany; 2 Department of Toxicology, University Medical Center, Johannes Gutenberg-University Mainz, 55131 Mainz, Germany; 3 Center for Thrombosis and Hemostasis (CTH), University Medical Center, Johannes Gutenberg-University Mainz, Langenbeckstrasse 1, 55131 Mainz, Germany; 4 German Center for Cardiovascular Research (DZHK), Partner Site Rhine-Main, University Medical Center, Johannes Gutenberg-University Mainz, 55131 Mainz, Germany

**Keywords:** Cancer metabolism, Cancer microenvironment

In their recent study published in *Nature*, Yuan and colleagues enlighten the role of histone lysine crotonylation (Kcr) in brain tumor biology. They report a novel lysine-dependent mechanism through which glioblastoma stem cells modulate type I interferon (IFNα/IFNβ) signaling to create an immunosuppressive environment.^1^

Glioblastoma (GBM) is the most common primary malignant tumor of the brain, exhibiting very poor prognosis with median overall survival of only about 12 months.^2^ State-of-the-art treatment includes surgery followed by radio-chemotherapy (typically with the DNA-damaging drug temozolomide). However, the aggressive character and the specialized immune microenvironment of GBMs often prevent satisfactory treatment success, resulting in tumor relapse and poor outcome.^3^ Thus, new therapeutic strategies that consider the heterogeneity and plasticity of GBM are urgently needed.

The posttranslational modification Kcr influences the fate of neuronal stem cells by affecting the chromatin microenvironment and transcriptome remodeling.^4^ Yuan et al. noted that glioblastoma stem cells expressed high levels of the transporter protein solute carrier family 7 member 2 (SLC7A2) to accumulate lysine for the generation of crotonyl-coenzyme A. This involves a reprogrammed lysine catabolism. The authors showed that the glutaryl-coenzyme A dehydrogenase (GCDH) pathway elevated Kcr predominantly at histone H4 (H4K5cr, H4K8cr, and H4K12cr), while down-regulating the crotonyl-coenzyme A hydratase enoyl-CoA hydratase short chain 1 (ECHS1). The addition of L-lysine elevated intracellular crotonyl-CoA levels in GBM stem cell cultures depending on functional GCDH. The fact that GCDH localized to the nuclear compartment by a low, but significant proportion, indicates that lysine catabolism takes part in nuclear processes. Immunoprecipitates of FLAG-tagged GCDH were found to contain the histone acetyltransferase CBP. Overexpression of CBP, but not of the related enzyme p300, increased H4Kcr in human embryonic kidney 293T cells. In vitro experiments disclosed that this enzymatic activity of CBP required the presence of GCDH converting crotonyl-CoA. Hence, CBP exerts biochemical activities as transferase of acetyl- and crotonyl-residues. At the cellular level, crotonate supplementation or ECHS1 depletion increased histone Kcr and repressed senescence in differentiated GBM cells.

Gene set enrichment analysis (GSEA) and Gene Ontology analysis revealed that genes linked to IFNα/IFNβ signaling were increased when GCDH was knocked down in glioblastoma stem cells. This was associated with proliferation arrest. The functional involvement of GCDH in the modulation of such cytokine signaling was confirmed through a site-directed mutagenesis approach. Catalytically compromised GCDH in cultured glioblastoma stem cells blocked its function in regulating type I IFN signaling. Although Kcr influenced epigenetic patterns by affecting H3K27ac and H3K9me3, changed interferon signaling was not a cause of altered lysine-catabolism-repressed genes. Instead, IFN signaling was a result of a local loss of histone Kcr together with an augmented acetylation of histone H3 K27 at repetitive elements. The resulting transcription of transposable retrotransposons drove cytosolic dsRNA and retrotransposon DNA generation, and triggered MDA5- and cGAS-dependent IFN signaling in glioblastoma stem cells. The inhibition of reverse transcriptase with the HIV drug zidovudine attenuated these beneficial processes.

By in silico analyses and ChIP-sequencing, MYC was identified as the transcription factor responsible for the perturbation of differentially expressed genes. The authors compared glioblastoma stem cells with differentiated glioblastoma cells and neural stem cells. Both, correlative and functional evidence link *MYC* expression to *GCDH* expression and histone Kcr protein levels in glioblastoma stem cells. In line with the tumor-suppressive roles of IFNs, the disruption of lysine catabolism and the consequential activation of IFN signaling was associated with a decreased glioblastoma stem cell proportion in intracranial tumors.^4^

When the authors reduced serum and tumor tissue lysine levels in healthy or tumor-bearing NOD.SCID γc-deficient mice with a lysine-restricted diet, MYC inhibition by the small molecule MYCi975 in combination with this diet improved their survival and diminished tumor cell counts. Furthermore, in a patient GBM dataset, the lysine-catabolism-repressed signature negatively correlated with *GCDH* mRNA levels and showed a positive correlation to immune cell signatures. Of note, this dietary phenotype also translated promisingly into immune checkpoint inhibitor therapy, where anti-PD-1 treatment of mice bearing intracranial tumors at day 21 post transplantation displayed elevated counts of CD8-positive T cells under lysine-restricted diet conditions.

**Fig. 1:**
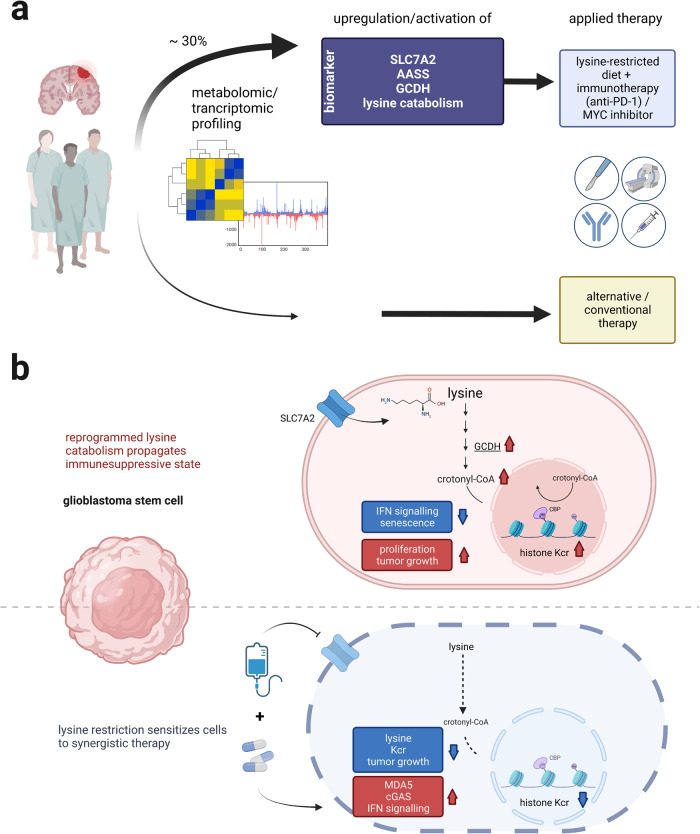
Reprogrammed lysine catabolism as intervention point for precision medicine in glioblastomas. **a** By metabolomic and/or transcriptomic profiling, glioblastoma (GBM) patients could be stratified for a clinical study evaluating the benefit of a combined lysine-restricted diet and immune/chemotherapy (such as anti-PD-1 / MYC inhibitor). Patients exhibiting a specific profile of one or more markers for reprogrammed lysine catabolism (expected around 30%), such as upregulation of the amino acid transporter SLC7A2 (Solute Carrier Family 7 Member 2), and enzymes of lysine catabolism (AASS, Aminoadipate-Semialdehyde Synthase; GCDH, Glutaryl-CoA Dehydrogenase), are promising candidates for application of the treatment aiming at sensitizing tumor cells to synergistic treatment by lysine restriction (see **b**). **b** Proposed mechanism underlying the application of a lysine-restricted combined immune/chemotherapy. Glioblastoma stem cells (GSC) gain an immunesuppressive state by reprogrammed lysine catabolism resulting in increased histone crotonylation (Kcr), and thus altered gene expression (upper panel). Consequently, limited availability of lysine has the potential to disrupt the proposed lysine-Kcr pathway, and thereby can sensitize GSCs to synergistic anti-tumor therapy (lower panel). Created with BioRender.com

At present, no inhibitors of GCDH are known. Such agents may become precious therapeutics and tools to dissect the biology of crotonylation. Concerning the impact of CBP on IFN signaling, this is reminiscent of the control of STAT1 signaling by CBP. Acetylation of STAT1 attenuates IFN-dependent signaling, and like its impact on crotonylation, this is specifically exerted by CBP and not by p300.^5^ Future research can show if there is an interplay between these activities of CBP and how this affects GSCs. Whether anti-HIV drugs affect GBM development and prognosis, is also a provocative question. The detailed characterization of a reprogrammed lysine catabolism resulting in histone crotonylation-dependent decrease of type I IFN signaling provides an attractive new “point of attack” to fight GBM by a combined lysine-restricted diet with synergistic immune/chemotherapy. Thus, the study gives the essential prerequisites for future clinical studies evaluating the benefit of the proposed combination therapy (Fig. 1). Although lysine restriction has been already suggested as therapeutic option for the treatment of other diseases, such as epilepsy, therapeutic interventions have to be monitored carefully in clinical settings. Due to the multiple physiological functions of non-histone protein crotonylation, an altered balance of the donor crotonyl-CoA might result in undesirable side effects.

Taken together, Yuan et al. provide valuable insights into a novel mechanism leading to an immunosuppressive state in glioblastoma stem cells. Future clinical studies addressing these challenges are urgently needed to assess the translational potential of the described mechanism as possible treatment option for GBM patients.
